# Biodegradation of fipronil: Molecular characterization, degradation kinetics, and metabolites

**DOI:** 10.21203/rs.3.rs-2885549/v1

**Published:** 2023-06-06

**Authors:** Anjali Jaiswal, Animesh Tripathi, Suresh Kumar Dubey

**Affiliations:** Molecular Ecology Laboratory, Department of Botany, Institute of Science, Banaras Hindu, University, Varanasi, Uttar Pradesh-221005, India

**Keywords:** Bioremediation, Degradation kinetics, Fipronil, Metabolites, 16S rRNA sequencing

## Abstract

Fipronil (C_12_H_4_Cl_2_F_6_N_4_OS), is a commonly used insecticide effective against numerous insects and pests. Its immense application poses harmful effects on various non-target organisms as well. Therefore, searching the effective methods for the degradation of fipronil is imperative and logical. In this study, fipronil-degrading bacterial species are isolated and characterized from diverse environments using a culture-dependent method followed by 16S rRNA gene sequencing. Phylogenetic analysis showed the homology of organisms with *Acinetobacter* sp., *Streptomyces* sp., *Pseudomonas* sp., *Agrobacterium* sp., *Rhodococcus* sp., *Kocuria* sp., *Priestia* sp., *Bacillus* sp., *Pantoea* sp. The bacterial degradation potential for fipronil was analyzed through High-Performance Liquid Chromatography. Incubation-based degradation studies revealed that *Pseudomonas* sp. and *Rhodococcus* sp. were found to be the most potent isolates that degraded fipronil at 100 mg L^−1^ concentration, with removal efficiencies of 85.97 % and 83.64 %, respectively. Kinetic parameter studies, following the Michaelis-Menten model, also revealed the high degradation efficiency of these isolates. Gas Chromatography-Mass Spectrometry (GC-MS) analysis revealed fipronil sulfide, benzaldehyde, (phenyl methylene) hydrazone, isomenthone, etc., as major metabolites of fipronil degradation. Overall investigation suggests that native bacterial species isolated from the contaminated environments could be efficiently utilized for the biodegradation of fipronil. The outcome derived from this study has immense significance in formulating an approach for bioremediation of fipronil-contaminated surroundings.

## Introduction

Fipronil [5-amino-1-[2,6-dichloro-4-(trifluoromethyl)phenyl]-4-[(trifluoromethyl)sulfinyl]- 1H-pyrazole-3-carbonitrile] is a systemic insecticide grouped under the phenylpyrazole family (Tomlin 2000; Tingle 2003). It is a broad-spectrum, among the most hazardous, lipophilic, and persistent pesticides used against rice stem borer, bollworm, ticks, aphids, locusts, termites, mosquitoes, ants, cockroaches, etc (Mohapatra et al. 2010). In recent years, it was estimated that the worldwide pesticide market dominates ~ 30% for the combination of neonicotinoid and fipronil together (Casida and Durkin 2013; Pang et al. 2020). Fipronil is considered as a “next-generation insecticide” because its mechanism mode is different from the conventional biochemical routes of previously known insecticides which include pyrethroids (sodium channel blockers), carbamates, and organophosphates (cholinesterase inhibitors), to which many insects/pests have evolved resistance (Bobe et al. 1997; Aajoud et al. 2003; Bhatti et al. 2019). Fipronil induces its toxicity on gamma-aminobutyric acid (GABA) receptors and acts as a nerve transmitter in insects. It blocks the passage of chloride ions through the GABA receptors which leads to the interruption in neuron signaling and finally closure of the central nervous system (CNS). It causes paralysis and eventually death of insects (Bhatti et al. 2019). The wider application, longer half-life (~ 3-7 months in field environment), and inappropriate management causes its augmentation in the environment that is detrimental to non-target biota (Bonmatin et al. 2015). WHO has also classified fipronil as a class II moderately hazardous pesticide. Therefore, removal of fipronil from a contaminated environment is of utmost importance.

Bioremediation is considered a cost-effective mechanism for the removal of harmful compounds like fipronil as microbial organisms play a crucial role in achieving biodegradation pathways of fipronil from a contaminated habitat (Li et al. 2012; Paliwal et al. 2015). There are many studies have been documented that incorporated fungal and bacterial isolates for the degradation of fipronil some of which have been shown here. Abraham and Gajendiran (2019) reported that *Streptomyces rochei* AJAG7 showed degradation efficiency of fipronil at 500 mg L^−1^ in MSM and soil in 6 and 7 days respectively. Fungal species such as *Trametes versicolor* and *Aspergillus glaucus* have been reported for their degradation potential of fipronil (Wolfand et al. 2016; Gajendiran and Abraham 2017). Bhatt et al. (2020) reported that *Bacillus* sp. strain FA3 degraded ~77% fipronil after 15 days of incubation period in MSM media and ~77.5% fipronil in soil. Bhatt et al. (2021) conducted another study for the biodegradation of fipronil and identified a strain FA4 as *Bacillus* sp. that can degrade fipronil up to 75% in MSM broth and 77% in soil. Sayi et al. (2020) identified a bacterial strain SNCK-4 named *Klebsiella pneumoniae* that can grow on a medium containing 1% fipronil as a carbon source. Thirumalaiselven et al. (2015) isolated bacterial isolates from a freshwater environment belonging to *Bacillus* sp. and *Comamonas aquatica*, and showed their potential to degrade 10.46 to 94.66% of the initial concentration fipronil of 10 and 20 mg L^−1^. Prado et al. (2021) reported the highest degradation (94%) by *Bacillus megaterium* strain E1 for fipronil (600 mg L^−1^) utilizing it as a solitary nitrogen and main carbon source. In a recent study, Viana et al. (2022) reported a strain RFD1C as *Bacillus amyloliquefaciens*, which achieved 93% degradation of 10 mg L^−1^ of fipronil during 5 days of incubation period. *Bacillus firmus, Burkholderia thailendensis, Acinetobacter calcoaceticus, Acinetobacter oleivorans, Paracoccus sp.,* Gamma Proteobacteria are some of the known bacterial isolates, involved in biodegradation of fipronil (Kumar et al. 2012; Mandal et al. 2014; Uniyal et al. 2016b; Cappelini et al. 2018). These studies reflected the considerable microbial potential for the degradation of fipronil.

In the contaminated surroundings, fipronil produces four major degradation products consisting of fipronil sulfide, fipronil sulfone, fipronil desulfinyl, and fipronil amide by the process of reduction, oxidation, photolysis, and hydrolysis, respectively (Gunasekara et al. 2007). These metabolites are also bioactive compounds and hazardous for many off-target organisms, which include butterflies, moths, pollinators (bees, bumblebees), and earthworms (Pisa et al. 2014; Bonmatin et al. 2015). However, few metabolites that are much more hazardous than the fipronil itself, may be similarly degraded with the help of using microbes (Masutti and Mermut 2007; Tan et al. 2008). Fipronil sulfone, fipronil sulfide, fipronil amide, Sulfurous acid, 2-ethylhexyl isohexyl ester, 1,2-benzene dicarboxylic acid, mono(2-ethylhexyl) ester, N-Phenylmethacrylamide, and Benzaldehyde, (phenyl methylene) hydrazone, have also been reported as biodegradation byproducts of fipronil (Mandal et al. 2013; Uniyal et al. 2016a; Abraham and Gajendiran 2019; At et al. 2019; Bhatt et al. 2021).

Due to wider applicability and longer persistence, the residual amount of fipronil reaches to various environmental components such as agricultural fields, sewage, and sewage treatment plant sludge. Over the course of time, the native bacterial species of these habitats get adapted to fipronil and develop capability for degrading it. It would be worthwhile to isolate bacterial species from such habitats and investigate their fipronil degradation efficacy. In context with this, in the present work, bacterial species from various fipronil-contaminated environments have been isolated and characterized and their efficacy for fipronil degradation has been determined. The process of fipronil degradation kinetics has been evaluated and their intermediate metabolites formed have also been detected.

## Materials and Methods

### Chemicals and media

Technical grade fipronil (analytical standard), with a purity of 95% was bought from Sigma-Aldrich, USA. HPLC grade acetonitrile, dichloromethane, and, ultra-pure HPLC water were purchased from E. Merck Limited, Mumbai, India. Acetonitrile and water were filtered through a 0.5 μm and 0.45 μm nitrocellulose syringe filter (Merck Millipore Ltd.) respectively before use. A stock solution of fipronil (1mg mL^−1^) was prepared in acetonitrile, filtered, and kept at 4 °C for further use (Gajendiran & Abraham 2017). For the fipronil biodegradation study, two different bacteriological grade media were used i.e., Mineral Salt Media (MSM) and Luria Bertani (LB) media. MSM was used for screening and isolation of fipronil-degrading bacterial strains with a composition of 9 g Na_2_HPO_4_, 1.5 g KH_2_PO_4_, 1g NH_4_Cl, 0.2 g MgSO_4_.7H_2_O, 1.2 mg Fe (III) [NH_4_] citrate, 20 mg CaCl_2_, 0.5 g NaHCO_3_ and 1 mL trace element solution (in 100 mL stock solution) of 50 mg FeSO_4_.7H_2_O, 1 mg ZnSO_4_.7H_2_O, 0.3 mg MnCl_2_.4H_2_O), 3 mg H_3_BO_3_, 2 mg CaCl_2_.6H_2_O, 0.1 mg CuCl_2_.2H_2_O, 0.2 mg, NiCl_2_.6H_2_O, 0.3 mg Na_2_MoO_4_.H_2_O per liter in double distilled water and pH was maintained between 6.8-7 (Maya et al. 2011, Singh et al. 2019). LB broth was used for the growth and sustenance of isolated bacterial strains. For preparing solidified media, 1.7% of bacteriological grade agar was added to the broth. For sterilization, both the media were autoclaved (121 °C, 20 min).

### Sample collection

Soil samples were collected from the Agricultural field, Banaras Hindu University, Varanasi (25°27’N, 83°01’E), samples of sludge from drainage (25°26’N, 82°99’E) and activated sludge sample from Sewage Treatment Plant (STP) Bhagwanpur, Varanasi, India (25°17’N, 83°00Έ) 83m above mean sea level (MSL). The topsoil layer samples (20 cm depth) were collected with the help of a sterile corer. Sludge samples were collected with the help of a sterile spatula. All the samples were placed in sterile poly bags, and transported to the laboratory. The collected samples were dried, passed through a 2 mm mesh sieve to remove debris, and stored at 4 °C for further investigation.

### Enrichment and isolation of fipronil-degrading bacterial species

Enrichment of bacterial strains from all three different samples were done in MSM in the presence of fipronil. 5 g of each of the samples were spiked with 50 mg L^−1^ of fipronil in three different 250 ml Erlenmeyer flasks containing 45 mL of MSM media. All three flasks were incubated on a shaker (120 rpm) at 28±2 °C for 2 weeks. After the incubation period, 1 mL of suspension from each flask was put into their respective flasks containing 50 mL fresh MSM supplemented with 50 mg L^−1^ fipronil and incubated on a rotary shaker at 120 rpm at room temperature for overnight. Serial dilution of overnight samples with microbial growth was made from 10^−1^ to 10^−9^ and incubated in vials containing MSM and fipronil (50 mg L^−4^). Lower dilutions were again transferred into vials having 10 mL MSM and fipronil (50 mg L^−4^) and were incubated on a shaker at 120 rpm (28±2°C). The control included MSM, and fipronil but absence of bacterial inoculum. Vials showed turbidity as an indicator of bacterial growth. Thus, 100 μl of enriched samples from vials showing the best bacterial growth were spread on MSM agar plates supplemented with 50 mg L^−1^ fipronil (Bhatt et al., 2021). Plates were incubated at 28°C for 3-4 days. The morphologically distinct isolates were obtained by repetitive streaking on MSM agar plates with fipronil. Pure isolates were stored at 4 °C as well as stocked in glycerol at −80 °C.

### Biochemical and molecular assessment of isolated fipronil degrading bacterial species

For the biochemical assessment of isolated bacterial strains, the methodology was used as discussed in Bergey’s Manual of Determinative Bacteriology (Holt et al. 1994). Further, molecular characterization of bacterial strains was done through 16S rRNA nucleotide sequencing of genomic DNA. Overnight grown cultures of bacterial strains were used for the extraction of genomic DNA with the help of MasterPure^™^ complete DNA & RNA purification kit (Lucigen). After the extraction of DNA, quantification was done using a spectrophotometer (Nanodrop^™^ Technologies, Incorporated, Wilmington, DE, USA). Amplification of 16S rRNA gene (1.5 kb sized) was done in a Thermal cycler (My Cycler^™^, Bio Rad Laboratories, Inc., Australia) using universal primers 27 F’ 5’ - AGAGTTTGATCMTGGCTCAG-3’ (forward) and 1492R’ 5’ - TACGGYTACCTTGTACGACTT-3’ (reverse) in a reaction mixture including 10X Buffer, 10 mM dNTPs, 500 U *Taq* DNA polymerase, 100 ng of template DNA and volume was maintained up to 50 μL with miliQ. The Polymerase Chain Reaction (PCR) cycling parameters were initial denaturation at 94 °C for 5 min that follows by 30 cycles of denaturation at 94 °C (1 min), further annealing at 55 °C (1min), extension step at 72 °C (1.30 min), and the last extension step at 72 °C for 5 min, uphold at 4 °C. DNA bands were observed on 1% agarose gel for PCR-amplified 16S rRNA amplicon size. Purification of amplified PCR products was done using MinElute® PCR Purification Kit (Qiagen) and sequencing was done by an automated sequencer at Centyle Biotech Pvt. Ltd., New Delhi using Sangers dideoxy nucleotide chain termination method. Processing of obtained sequence was done by BioEdit Sequence Alignment Editor (Version 7.2.5). Further identification of sequences was done based on reference species present in National Center of Biotechnology Information (NCBI) using BLAST (Basic Local Alignment Search Tool). Finally, the sequences were submitted with NCBI database to get the GenBank Accession number. The phylogenetic tree was constructed for bacterial strains by the neighbor-joining method using MEGA X (Version 11.0.13) software (Prado et al. 2021).

### Biodegradation of fipronil

To check the tolerance capacity of bacterial strains, a gradually increasing concentration range of fipronil was selected in which bacteria showed enormous growth in a short period. The inoculum for batch culture studies was prepared through LB overnight culture of bacterial strains. Cells were then centrifuged (5000 rpm; 15 min), pellets were washed with sterile saline (0.85%) and resuspended in sterile saline. A series of 100 ml flasks comprising 50 ml MSM were inoculated with 2 mL inoculum of individual isolates and supplemented with gradually increasing fipronil concentrations of 10, 25, 50, 100, 200, 300, & 400 mg L^−1^. Flasks in the absence of bacteria were considered as control for the comparison of bacterial growth. These were incubated at 28±2 °C at 120 rpm for 13 days and growth was monitored regularly with the help of a spectrophotometer (OD_600_). All these experiments were performed in replicates to minimize the chances of error. In order to determine the degradation potential of bacterial strains for fipronil, HPLC analysis was performed.

For the residue analysis of fipronil in samples, HPLC was performed through a method described by Abraham and Gajendiran (2019). For fipronil residue extraction, after 13 days of incubation period, 10 ml samples from each flask were withdrawn in a separate centrifuge tube and equal volume of 10 mL of dichloromethane was added to them. All the tubes were shaken vigorously for 5 min and kept aside in order to get a clean separation of phases. 5 ml of dichloromethane layer was collected in a separate tube and evaporated in a vacuum evaporator and it was resuspended in an equal volume of acetonitrile. Extracted fipronil concentration was analyzed by Waters (*717*plus Autosampler) HPLC (Waters Corporation, Milford, USA) equipped with reverse phase C18 column (SunFire® 4.6 mm × 250 mm × 5 μm) and Photodiode Array (PDA) detector (Waters 2998). Acetonitrile and water (80:20, v/v) including 0.1% phosphoric acid were used as a mobile phase, and the flow rate was maintained at 1 mL min^1−^. 20 μL samples were injected and peaks were detected at a wavelength of 278 nm. All the samples were filtered through a 0.22 μm nylon syringe filter (Merck Millipore Ltd.) prior to their injection. For the quantification of fipronil concentration, a standard curve of peak area vs fipronil concentration was prepared. In order to know whether average degradation rate varied depending on concentration, the following formula (1) was used to obtain the average relative degradation rate of fipronil degradation at different concentrations:

(1) $$r_{avg}\left(d^{-1}\right)=\frac{C_{0}-C_{t}}{C_{0}\left(\Delta t\right)}$$

Where r_avg_ is average relative degradation rate, C_0_ is fipronil concentration at initial time, C_t_ is fipronil concentration at ‘t’ time, and Δt is incubation period. Further, the initial fipronil concentration which showed maximum degradation percent and average relative degradation rate (r_avg_) was chosen for the study of kinetic constants using the Michaelis-Menten model. MSM media containing individual isolates and amended with optimum fipronil concentration were incubated at 28±2 °C at 100 rpm for 13 days. For analyzing residue fipronil concentration using HPLC, samples were collected regularly at time intervals of 24 hr.

### Metabolites analysis of fipronil degradation by FTIR and GC-MS

To determine the intermediates produced during the biodegradation of fipronil, FTIR and GC-MS analyses were performed. Two of the most efficient bacteria from the above study were selected for this experiment. 100 mL flasks containing 40 mL sterilized MSM were inoculated with 2 ml of both of the active bacterial strains individually and spiked with the 50 mg L^−1^ concentration of fipronil. These were incubated for 7 days at 28±2 °C at 100 rpm. After centrifugation (5000 rpm 15 min) of bacterial culture, the supernatant was taken for further extraction of fipronil and its metabolites through liquid-liquid partitioning by adding an equal volume of dichloromethane and then evaporated on a rotary evaporator to concentrate the sample. Further, it was resuspended in 4 mL MS-grade acetonitrile and analyzed on a triple quadrupole Gas Chromatography Tandem Mass Spectrometry (GC-MS/MS TQ-8050 NX, Shimadzu, Japan) with autosampler /injector AOC-20i+s. Rxi®- 5 Sil MS Capillary column (5% diphenyl / 95% dimethyl polysiloxane; 30m, 0.25mm, 0.25μm) (Restek, USA) was used for separation. 1 μL sample was injected in a linear velocity, split mode with a ratio of 10, and the injector temperature was 260°C. Carrier gas, Helium was used with a flow rate of 1.21 mL min^−1^. The oven temperature program was as follows: 100 °C (2 min hold) and increased up to 300 °C at a rate of 10 °C min^−1^ (18 min hold). Ion source temperature was 220 °C and the interface temperature was 270 °C with a solvent cut time of 4.50 min and operated in Flame Thermionic Detector/ Barrier discharge Ionization Detector (FTD/ BID) mode. MS has performed in Multiple Reaction Monitoring (MRM) mode. Data were processed and integrated through Shimadzu Real-time analysis software. On the basis of retention time (RT) and molecular weight (m/z, 40-600), fipronil and its metabolites were identified using National Institute of Standards and Technology (NIST) library database.

For identification of functional group present in parent chemical and its intermediate metabolites FTIR analysis was performed through KBr pellet mode on Nicolet iS5 (THERMO Electron Scientific Instruments LLC). Spectra were recorded in the infrared region of 400-4000 cm^−1^ with a scan speed of 64 and resolution of 4 cm^−1^. For sample preparation, 20 mL sterilized MSM were enriched with fipronil (50 mg L^−1^) and 1 mL bacterial inoculum of both strains individually. A control sample was prepared without bacterial inoculum. After 7 days of the incubation period (28±2 °C, shaken at 100 rpm), the supernatant was collected by centrifugation (5000 rpm 15 min), and extraction of compounds was done by adding an equal volume of dichloromethane. The organic layer was evaporated on a rotary evaporator and residues were reconstituted in acetonitrile for analysis of the functional groups and any bond stretching due to fipronil biodegradation.

## Result and Discussion

### Biochemical and molecular assessment of isolated fipronil-degrading bacterial species

**FIG.1.**
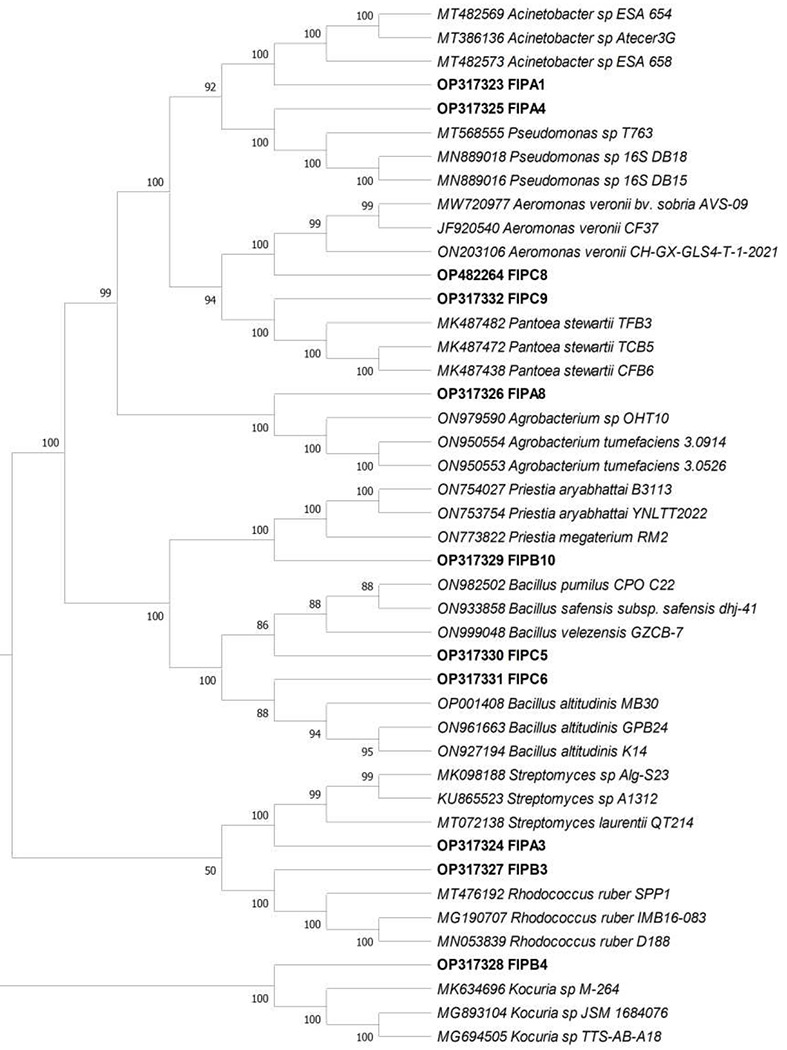
Phylogenetic tree for eleven isolates based on 16S rRNA nucleotide sequences constructed by the neighbor-joining method. Numerical values at the node represent bootstrap percentile values.

Identification of isolates were done on the basis of morphological, biochemical, and molecular characterization. Since each type of bacterial isolate holds unique enzymatic profiles, thus helps in the identification of particular ones. Eleven different bacterial isolates were obtained growing in the presence of fipronil, as identified on the basis of morphology. Out of eleven isolates, five (FIP_A1, FIP_A4, FIP_A8, FIP_C8, and FIP_C9) and six (FIP_A3, FIP_B3, FIP_B4, FIP_B10, FIP_C5, and FIP_C6) were gram negative and gram-positive, respectively. All the isolates showed positive responses for catalase test while in case of urease test, only three isolates (FIP_A3, FIP_A8, and FIP_B3) gave positive results. All the isolates except FIP_B3 and FIP_B4 showed positive results for citrate utilization test. For the oxidase test, FIP_A3, FIP_A4, FIP_A8, FIP_B4, and FIP_C8 were found positive except these all showed negative results. For the nitrate reduction test, only isolates FIP_A1, FIP_A3, FIP_A8, and FIP_C9 showed negative results. Biochemical test results of all isolates have been enlisted in **Table S1**. 16S rRNA nucleotide sequencing and phylogenetic tree analysis validated that isolate belonged to ten different bacterial genera *Acinetobacter* sp. (FIP_A1), *Streptomyces* sp. (FIP_A3), *Pseudomonas* sp. (FIP_A4), *Agrobacterium* sp. (FIP_A8), *Rhodococcus* sp. (FIP_B3), *Kocuria* sp. (FIP_B4), *Priestia* sp. (FIP_B10), *Bacillus* sp. (FIP_C5, FIP_C6), *Aeromonas* sp. (Fip_C8), *Pantoea* sp. (FIP_ C9) (Fig. 1).

Similar bacterial isolates have also been reported by other investigators for the biodegradation of fipronil. Abraham and Gajendiran (2019) published that *Streptomyces* sp. competent for degrading fipronil in aqueous media and soil. Prado et al. (2021) showed the highest fipronil degradation using *Bacillus megaterium. Acinetobacter calcoaceticus and Acinetobacter oleivorans* also showed their efficacy in degrading fipronil in soils (Uniyal et al. 2016b). Various species of *Bacillus* have also been showed for the degradation of fipronil (Mandal et al. 2013; Mandal et al. 2014; Bhatt et al. 2020; Bhatt et al. 2021; Gangola et al. 2021; Viana et al. 2022). *Rhodococcus* sp. was also reported for the degradation of many pesticides like endosulfan, cypermethrin, acetamiprid, and carbendazim, etc (Verma et al. 2006; Phugare and Jadhav 2015; Abraham and Silambarasan 2018; He et al. 2022). *Pseudomonas* sp. was also found to be a degrader of pesticides like imidacloprid, sulfoxaflor, and chlorpyrifos, etc (Pandey et al. 2009; Yadav et al. 2014; Jiang et al. 2022).

Processed 16S rRNA nucleotide sequences of eleven isolates were submitted to the NCBI gene database under accession numbers OP317323 to OP317332, and OP482264. Since *Aeromonas* sp. was found to be a pathogenic bacterium, it was not used for further study.

### Biodegradation of fipronil

**FIG.2.**
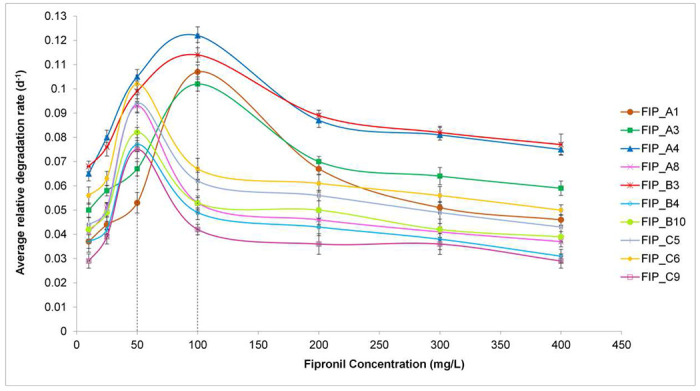
Average relative fipronil degradation rate (r_avg_) at 10-400 mg L^−1^ of fipronil concentration showing maximum degradation rate at 50 and 100 mg L^−1^. Data are depicted in mean ± standard deviation from triplicate values.

The results of average degradation rate of fipronil degradation studies by bacterial isolates are shown in Fig. 2. The relative average rate of fipronil degradation was increased up to 100 mg L^−1^ for bacterial isolates FIP_A1, FIP_A3, FIP_A4, and FIP_B3 and increased up to 50 mg L^−1^ for FIP_A8, FIP_B4, FIP_B10, FIP_C5, FIP_C6, and FIP_C9. There was a significant reduction in the biodegradation efficiency of bacterial isolates beyond these two optimum concentrations which could be due to the higher concentration of fipronil may be inhibitory to bacterial cells (Uniyal et al. 2016b). The variation in relative average degradation rates was followed in the range of 0.075 to 0.107 d^−1^. The highest value for the relative average degradation rate was observed in case of FIP_A4 (0.122 d^−1^), and FIP_B3 (0.114 d^−1^) at 100 mg L^−1^ while the lowest in case of FIP_C9 (0.075 d^−1^) at 50 mg L^−1^.

**FIG.3.**
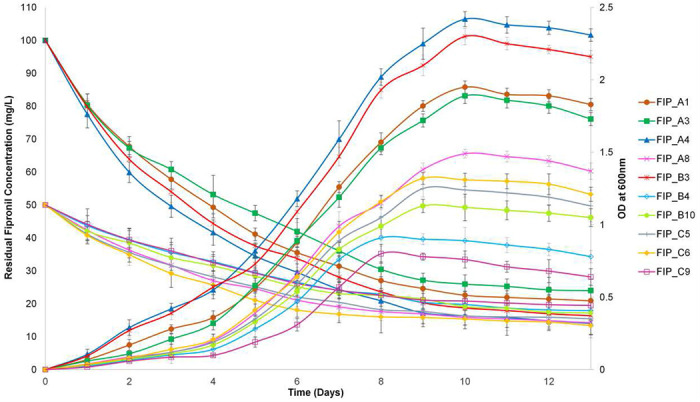
Residual fipronil concentration (mg L^−1^) on left Y-axis while the bacterial growth (OD) on right Y-axis is depicted at their respective optimum fipronil concentrations. The data are shown in mean ± standard deviation from triplicate experiments.

Growth of all the bacterial isolates and fipronil degradation studies were depicted in Fig. 3. Results obtained through batch culture studies showed that the growth curve followed an S-shaped graph for bacterial isolates FIP_A1, FIP_A3, FIP_A4, and FIP_B3 (100 mg L^−1^) and FIP_A8, FIP_B4, FIP_B10, FIP_C5, FIP_C6, and FIP_C9 (50 mg L^−1^) (Fig. 3). It was observed that growth of bacteria increased up to the tenth day (for FIP_A1, FIP_A3, FIP_A4, FIP_A8, FIP_B3), the ninth day (for FIP_C5, FIP_C6, FIP_B10) and eighth day (for FIP_B4, FIP_C9), then growth became stationary and decreasing due to paucity of nutrients and accumulation of toxic intermediates during the incubation period. Similarly, a reduction in the concentration of fipronil was observed up to the ninth day for isolates FIP_A1, FIP_A3, FIP_A4, and FIP_B3; the eighth day for isolates FIP_A8, FIP_B10, and seventh day for FIP_B4, FIP_C5, FIP_C6, FIP_C9. The degradation rate decreased and finally stabilized after 9 days as bacterial isolates entered their stationery and death phases, respectively. Degradation percent for all the bacterial isolates was observed within the range of 61-86%. Maximum degradation percent found after 13 days’ time period were 85.97 and 83.64% at 100 mg L^−1^ of fipronil concentration, in the case of FIP_A4 and FIP_B3 respectively.

A bacterial strain *Stenotrophomonas acidaminiphila* was reported by Uniyal et al. (2016a), which was able to degrade 86.14% of 25 mg L^−1^ fipronil in 14 days as a carbon source. A study reported by At et al. (2019) showed that *Staphylococcus arlettae* and *Bacillus thuringiensis* were able to degrade 76.39% and 65.95% of 10 mg L^−1^ of fipronil after 7 days. These results were obtained at relatively lower concentrations in contrast to the present study. While Gangola et al. (2021) performed a degradation study at relatively higher concentration and obtained a bacterial isolate 2D named Bacillus cereus, degraded 89% fipronil (450 ppm) in 15 days of incubation period in MSM medium. The results obtained through the present investigation are in line with previous reports for fipronil biodegradation as these isolates have potential to utilize it as a sole carbon source and energy. Many investigators also showed ability of bacterial isolates to fulfill their nutritional requirements by consuming fipronil as a carbon and energy source (Mandal et al. 2013; Bhatt et al. 2021; Abraham and Gajendiran 2019).

### Biodegradation kinetics of fipronil

The kinetics of fipronil biodegradation at the optimum concentration was investigated by selecting the Michaelis-Menten paradigm of the microbial kinetics model because substrate concentration and oxygen limitation did not serve as inhibitory factors in the biodegradation process. For the biodegradation process, kinetic parameters were resolved as described by Gautam and Dubey (2022). The non-linear kinetic model for the Michaelis-Menten equation is as follows (Eq.2):

(2) $$\frac{dS}{dt}=-V_{\max}\frac{S}{S+K_{s}}$$

**FIG.4.**
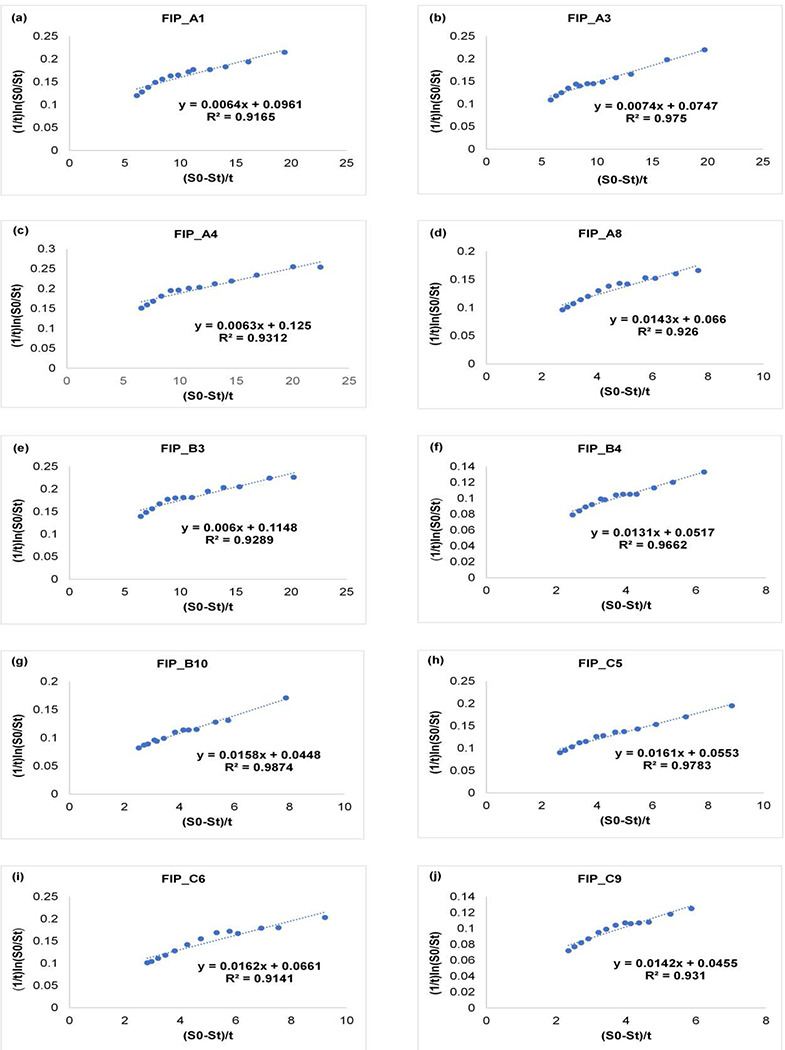
Fipronil biodegradation kinetics estimation using Lineweaver Burk equation for analyzing Michaelis Menten paradigm for microbial isolates: **(a)** FIP_A1 **(b)** FIP_A3 **(c)** FIP_A4 **(d)** FIP_A8 **(e)** FIP_B3 **(f)** FIP_ B4 **(g)** FIP_B10 **(h)** FIP_C5 **(i)** FIP_C6 **(j)** FIP_C9.

Kinetic parameters were calculated with the help of the Lineweaver Burk equation, also known as the double reciprocal plot by linear regression plot (Eq.3).

(3) $$\frac{1}{t}ln\frac{S_{0}}{S_{t}}=\frac{S_{0}-S_{t}}{K_{s}t}+\frac{V_{\max}}{K_{s}}$$

where t is time (day), V_max_ is maximum degradation rate of substrate (mg L^−1^ d^−1^), S_0_ and S_t_ are substrate (fipronil) concentrations (mg L^−1^) at time 0 and t respectively, K_s_ is Michaelis-Menten constant (mg L^−1^) (replaced K_m_ from original equation) because biodegradation activity is accessed by virtue of intact bacterial cells instead of purified enzymes. K_s_ is half-saturation concentration of substrate or the concentration of substrate at which reaction achieves half of its V_max_ (maximum degradation rate). Values of K_s_ and V_max_/K_S_ were deduced through slope and intercept of the Lineweaver Burk equation respectively. 1/t (ln S_0_/S_t_) was plotted against (S_0_-S_t_)/t to obtain these kinetic parameters, K_s_ was calculated through the inverse of slope and V_max_ was obtained through intercept of the best fit straight line of experimental data. V_max_/K_S_ was calculated after getting the values of K_s_ and V_max_ (Singh et al. 2019). The biodegradation kinetics of fipronil followed a first-order reaction (Bhatt et al. 2020; Bhatt et al. 2021) because, in the natural environment, bacterial inoculum (enzyme) was comparatively higher than the substrate (fipronil) concentration that is required to be degraded. Fipronil uptake efficiency of bacterial isolates can be demonstrated by lower values of K_s_ and higher values of V_max_ but V_max_/K_s_ (specific substrate affinity) can be considered as a better parameter instead of V_max_ and K_s_ individually for measuring nutrient (fipronil) uptake efficiency and assimilation ability of bacterial isolates (Silambarasan and Vangnai 2016). Higher the values of V_max_/K_s_, the higher will be the fipronil utilization or degradation potential and growth of bacterial strains. The degradation kinetics of fipronil by all the bacterial isolates unveiled conformity with the Lineweaver Burk plot (linearized form) as depicted in Fig. 4 and the values of V_max_, K_s_, and, V_max_/K_s_ for all the bacterial isolates have been represented in Table 1.

Results obtained through our present study indicated that the value of K_s_ (mg L^−1^) was found between 135.13 to 166.67 mg L^−1^ for isolates FIP_A1, FIP_A3, FIP_A4, FIP_B3, and between 61.73 to 76.33 mg L^−1^ for isolates FIP_A8, FIP_B4, FIP_B10, FIP_C5, FIP_C6, FIP_C9. However, values of V_max_ (mg L^−1^ d^−1^) were found highest for FIP_A4 (19.84) and FIP_B3 (19.13) followed by isolates FIP_A1 > A3 > A8 > C6 > B4 > C5 > C9 > B10 values ranged between 2.83 to 15.01 mg L^−1^ d^−1^. The values of V_max_/K_s_ (d^−1^) were found highest for isolates FIP_A4 and FIP_B3 i.e., 0.125 and 0.115 respectively whereas it was between 0.045 to 0.096 for the remaining strains with trend FIP_A1 > FIP_A3 > FIP_A8 = FIP_C6 > FIP_C5 > FIP_B4 > FIP_B10 = FIP_C9. The result obtained through the above kinetic study showed that the highest V_max_/K_s_ was observed in the case of FIP_A4 and FIP_B3, followed by other eight bacterial isolates. On that basis, it was concluded that *Pseudomonas* sp. FIP_A4 and *Rhodococcus* sp. FIP_B3 was the most efficient degrader of fipronil while the isolate *Pantoea* sp. FIP_C9 was found to be the least efficient among all the ten bacterial isolates.

Bhatt et al. (2020) reported that *Bacillus* sp. FA3 followed first-order reaction kinetics for degradation of 50 mg L^−1^ of fipronil concentration with a degradation constant (k) of 0.0891 day^−1^, half-life (t_1/2_) of 7.7 days, determination coefficient (R^2^) of 0.921 day^−1^ and Ks of 65.096 mg L^-1^. Bhatt et al. (2021) conducted another study for understanding the biodegradation kinetics of fipronil (50 mg L^−1^) and reported that *Bacillus* sp. FA4 followed the kinetics of first-order reaction for fipronil biodegradation with k, t_1/2_, R^2^, and K_s_ of 0.0861 day^−1^, 8.04 days, 0.970 days^−1^, and 12.08 mg L^−1^. However, sufficient data for comparison was unavailable related to the kinetic parameters of the Lineweaver Burk plot due to the lack of Michaelis-Menten kinetic study on fipronil biodegradation. Therefore, comparison of all the kinetic parameters was not discussed here. Gautam and Dubey (2022) reported kinetic parameters for the biodegradation of imidacloprid, a neonicotinoid insecticide i.e., K_s_ values ranged between 70.9 to 144.9 mg L^−1^, V_max_ values ranged between 3.7 to 15.5 mg L^−1^ d^−1^ and V_max_/K_s_(d^−1^) values ranged from 0.051 to 0.107. Subsanguan et al. (2020) reported the kinetics results for the biodegradation of profenofos, an organophosphorus insecticide, the K_s_, V_max_, and V_max_/K_s_ values found 92.07, 13.07, and 0.14 respectively. Based on the above result it was concluded that the result of present study was in line and showed conformity with other previous results.

### Metabolites analysis of fipronil

**FIG 5:**
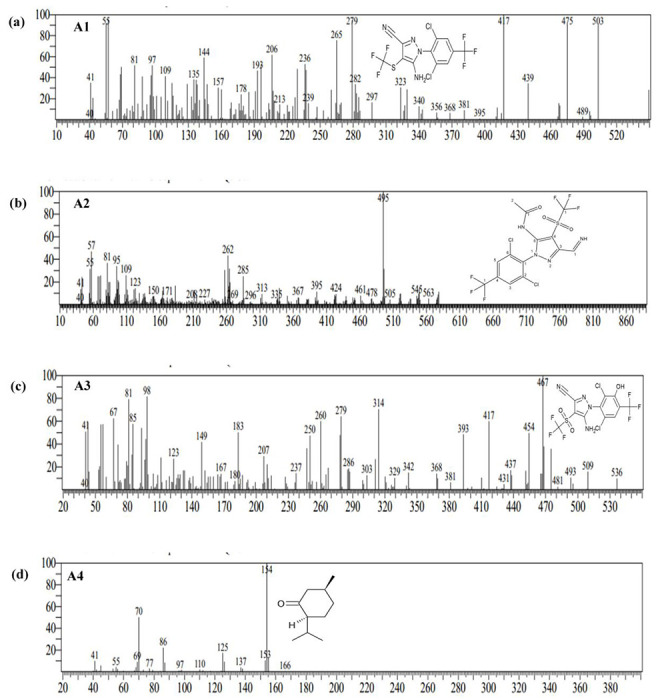
The mass spectra of metabolites obtained by GC-MS analysis during biodegradation of fipronil by strain FIP_A4 **(a)** Fipronil sulfide **(b)** N-[2-(2,6-Dichloro-4-trifluoromethyl-phenyl)-5-iminomethyl-4-trifluoromethanesulfonyl-2Hpyrazol-3-yl]-acetamide **(c)** Hydroxylated fipronil sulfone **(d)** Isomenthone.

**FIG 6:**
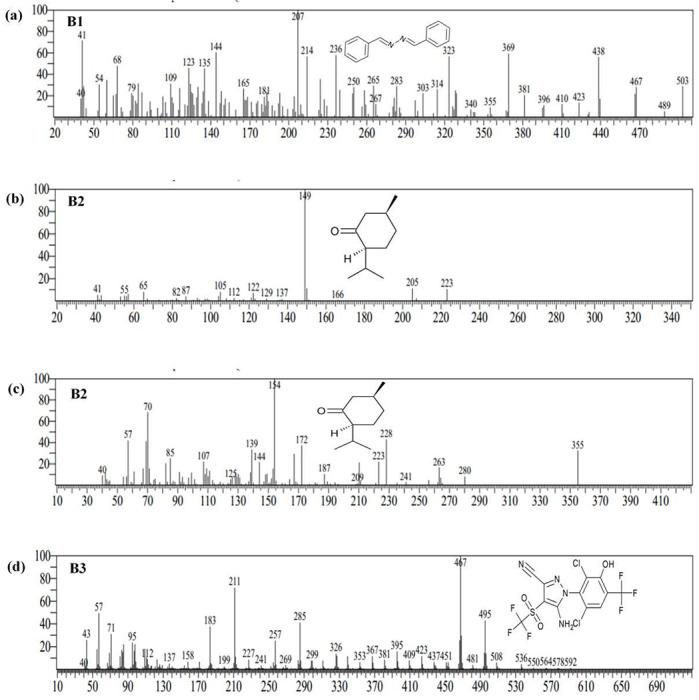
The mass spectra of metabolites obtained by GC-MS analysis during biodegradation of fipronil by strain FIP_B3 **(a)** Benzaldehyde, (phenylmethylene) hydrazone **(b)** Isomenthone **(c)** Isomenthone **(d)** Hydroxylated fipronil sulfone.

Since bacterial isolates FIP_A4 and FIP_B3 were identified as the most efficient degrader of fipronil as their specific substrate affinity (V_max_/K_s_) was found highest, metabolites formed by them were analyzed. Metabolome analysis using GC-MS provided information regarding the compounds formed after the degradation of fipronil by FIP_A4 and FIP_B3 **(Fig. S1)**. For FIP_A4 compound A1 was identified as fipronil sulfide at retention time (RT) 28.645 min with m/z of 417.51, compound A2 as N-[2-(2,6-Dichloro-4-trifluoromethyl-phenyl)-5-iminomethyl-4-trifluoromethanesulfonyl-2Hpyrazol-3-yl]-acetamide at RT 32.945 min with m/z of 495.45. The compound A3 was identified at RT 24.950 min with m/z of 467.40 as hydroxylated fipronil sulfone and the compound A4 at RT 13.8 min with m/z of 154.10 was identified as isomenthone (Fig. 5). While samples inoculated with FIP_B3 revealed the production of three compounds i.e., compounds B1, B2, and B3 that were characterized as benzaldehyde, (phenylmethylene) hydrazone (RT at 25.905 min), isomenthone (RT at 14.082 and 16.940 min) and hydroxylated fipronil sulfone (RT at 30.082min) with m/z ratio of 207.05, 149.05, 154.10 and 467.45 respectively (Fig. 6). All metabolites have been enlisted in Table 2. In comparison to the chromatogram of control, fipronil peak area and concentration were found less in both the bacterial-treated samples, these were the result of bacterial degradation of fipronil. The concentration of produced intermediate metabolites was found less in comparison to the parent chemical.

Formation of the initial transformed product of fipronil i.e., fipronil sulfone and fipronil sulfide occurs via oxidation and reduction mechanisms respectively (Uniyal et al. 2016b; Cappelini et al. 2018; Bhatti et al. 2019). Similar metabolites such as isomenthone, and benzaldehyde, (phenyl methylene) hydrazone were identified during the biodegradation of fipronil by a bacterium *Streptomyces rochei* strain AJAG7 and a fungus *Aspergillus glaucus* strain AJAG1 respectively (Gajendiran and Abraham 2017; Abraham and Gajendiran 2019). A compound named N-[2-(2,6-Dichloro-4-trifluoromethyl-phenyl)-5-iminomethyl-4-trifluoromethanesulfonyl-2Hpyrazol-3-yl]-acetamide was detected in a microbial fuel cell in the process of degradation of fipronil (Zhang et al. 2019). For the fungal degradation of fipronil, an intermediate metabolite named hydroxylated fipronil sulfone was detected and reported by Wolfand et al. (2016). Based on the identified metabolites it can be proposed that fipronil can be metabolized into fipronil sulfide and fipronil sulfone by reduction and oxidation reactions respectively. Since fipronil sulfone is an unstable compound, a hydroxylation reaction occurs leading to the formation of hydroxylated fipronil sulfone. Further, it may be converted into other simpler forms of metabolites which have been reported. Overall, from the above findings, it can be deduced that highly toxic fipronil can be metabolized into their simpler and less toxic intermediate compounds with the help of these bacterial species.

The biodegradation of fipronil is confirmed by correlating the numerous changes in FTIR spectra of bacterial strain-treated samples (MSM + Fipronil + bacterial inoculum) with control (MSM + Fipronil) **(Fig. S2)**. A peak at 1735 cm^−1^ was observed in the samples treated with bacterial strains which correspond to ester groups indicating the formation of esters upon degradation of fipronil. Another band in treated samples at 1221cm^−1^ also indicates formation of ester group in the samples. Peak formed around 1350 cm^−1^ showed the formation of sulfone derivatives of fipronil further proving the degradation of fipronil to its sulfone derivatives.

Gajendiran and Abraham (2017) in fipronil degradation by *Aspergillus glaucus* strain AJAG1 observed similar bands in FTIR spectra around 1737 and 1271 cm^−1^ indicating formation of esters after degradation of fipronil. Control and bacterial-treated samples both include some similar peaks which could be due to the undegraded fipronil. Singh et al. (2021) reviewed formation of various sulphone derivatives of fipronil after biodegradation and hence provides support to our analysis.

## Conclusions

Bacterial strains isolated from fipronil-contaminated environments showed high potential for removal of fipronil (10-400 mg L^−1^ concentration) with degradation efficiency of ~ 61 to 86 % in 13 days. Among all the isolated bacterial species *Pseudomonas* sp. FIPA4 and *Rhodococcus* sp. FIPB3 were found to be the most promising for the bioremediation of fipronil. Their higher values for kinetic parameters such as V_max_ and V_max_/K_s_ also ratified the efficiency of these two isolates for the removal of higher concentrations of fipronil. Identified intermediate metabolites provide evidence of bacterial degradation of fipronil. Thus, these isolates can aid in the degradation of fipronil and contribute to the reduction of its harmful effects on the environment and human health.

## Figures and Tables

**Table 1:** Percentage biodegradation (%), average relative degradation rate (r_avg_), kinetic parameters K_s_ (mg L^−1^), V_max_ (mg L^−1^ d^−1^), V_max_/K_s_ (d^−1^) for fipronil degradation by bacterial species. The data are depicted in mean ± standard deviation from triplicate experiments.

Parameters	Bacterial Isolates									
	FIP_A1	FIP_A3	FIP_A4	FIP_A8	FIP_B3	FIP_B4	FIP_B10	FIP_C5	FIP_C6	FIP_C9
% Degradation	79.08±2.9	75.66±1.9	85.97±1.8	71.52±2.2	83.64±2.08	64.14±2.4	65.54±2.4	69.1±2.06	73.18±1.9	61.02±2.5
r_avg_. (d^−1^	0.107	0.102	0.122	0.093	0.114	0.077	0.082	0.094	0.102	0.075
K_s_ (mg L^−1^)	156.25±2.3	135.13±2.4	158.73±3.8	69.93±2	166.67±2.6	76.33±2.07	63.29±2.3	62.11±2.6	61.73±2.2	70.42±2.2
V_max_ (mg L^−1^ d^−1^)	15.01±1.9	10.09±0.98	19.84±2.7	4.61±1.1	19.13±1.4	3.95±1.11	2.83±0.91	3.43±0.92	4.08±0.91	3.2±0.49
V_max_/K_s_ (d^−1^)	0.096	0.075	0.125	0.066	0.115	0.052	0.045	0.055	0.066	0.045

**Table 2.** Characteristics of metabolites produced during biodegradation of fipronil using bacterial species FIP_A4 and FIP_B3.

ID	Intermediate Metabolites	Mol. Wt. (g mol^−1^)	m/z ratio	Retention time (RT) (min)
A1	Fipronil sulfide	421.1	417.51	28.645
A2	N-[2-(2,6-Dichloro-4-trifluoromethyl-phenyl)-5-iminomethyl-4-trifluoromethanesulfonyl-2Hpyrazol-3-yl]-acetamide	497.119	495.45	32.945
A3	Hydroxylated fipronil sulfone	469.146	467.40	24.940
A4	Isomenthone	154.25	154.10	13.8
B1	Benzaldehyde, (phenylmethylene) hydrazone	208.26	207.05	25.905
B2	Isomenthone	154.25	149.05 & 154.10	14.082 & 16.940
B3	Hydroxylated fipronil sulfone	469.146	467.45	30.082

## Data Availability

Accession numbers (OP317323 to OP317332, and OP482264) of 16S rRNA nucleotide sequences for eleven bacterial isolates used in this study are available in NCBI gene database.
